# The serum oestradiol/progesterone ratio on the day of OPU + 7, but not the day of OPU + 5, affects the rates of live birth in fresh blastocyst embryo transfer cycles

**DOI:** 10.1186/s13048-023-01096-3

**Published:** 2023-01-07

**Authors:** Wenxian Zhao, Honglu Diao, Xin Chen, Shaoyuan Xu, Shengfang Jiang, Hong Cao, Changjun Zhang, Ying Zhang

**Affiliations:** 1 Reproductive Medicine Centre, Renmin Hospital, Hubei University of Medicine, Shiyan, People’s Republic of China; 2 Hubei Clinical Research Centre for Reproductive Medicine, Shiyan, People’s Republic of China; 3 Biomedical Engineering College, Hubei University of Medicine, Shiyan, People’s Republic of China; 4 Biomedical Research Institute, Hubei University of Medicine, Shiyan, People’s Republic of China; 5 Hubei Key Laboratory of Embryonic Stem Cell Research, Hubei University of Medicine, Shiyan, People’s Republic of China; 6 Department of Orthopaedic Surgery, Renmin Hospital, Hubei University of Medicine, Shiyan, People’s Republic of China

**Keywords:** Luteal phase, Hormones, Progesterone, Estradiol, In vitro fertilization

## Abstract

**Background:**

In an in vitro fertilization (IVF) cycle, the embryo ends its wandering time and begins the process of implantation into the uterine cavity on the seventh day after oocyte pick-up (OPU + 7), which is closer than OPU + 5 to the time of nidation. Therefore, measuring the oestradiol (E2)/progesterone (P) ratio on OPU + 7 may be helpful for predicting pregnancy outcomes.

**Methods:**

This is a retrospective cohort study of 2,257 women undergoing a follicular-phase depot gonadotropin-releasing hormone agonist (GnRH-a) protocol for in vitro fertilization /intracytoplasmic sperm injection (IVF/ICSI) treatment and fresh blastocyst embryo transfer cycles at a university-affiliated fertility center between January 2016 and April 2021. First, 2,257 women were split into two groups based on clinical pregnancy for analyzing the levels of E_2_ and P and the E_2_/P ratio on the day of OPU + 2, OPU + 5 and OPU + 7. And then 2,257 cycles were stratified into three groups based on E_2_/P ratio tertiles on OPU + 7: the low group (1.3–15.7 pg/ng), middle group (15.7–28.8 pg/ng), and high group (28.8–487.2 pg/ng). The threshold effect of the E_2_/P ratio on OPU + 7 on live birth was investigated using a two-piecewise linear regression model and a smoothing function curve.

**Results:**

The level of P in the clinical pregnancy group were lower than that in the nonclinical pregnancy group on both OPU + 2 and OPU + 7 (201.9 ± 71.6 ng/ml vs 213.1 ± 77.6 ng/ml, 89.5 ± 88.5 ng/ml vs 99.5 ± 94.9 ng/ml, *P* < 0.05). The E_2_/P ratio in the clinical pregnancy group were higher than that in the nonclinical pregnancy group on both OPU + 2 and OPU + 7 (8.4 ± 6.5 pg/ng vs 8.0 ± 6.8 pg/ng, 32.3 ± 38.5 pg/ng vs 25.2 ± 31.0 pg/ng, *P* < 0.01). The E_2_/P ratio on OPU + 7 was positively associated with positive hCG (adjusted OR = 1.01; 95% CI, 1.01–1.02; *P* < 0.0001), clinical pregnancy (adjusted OR = 1.01; 95% CI, 1.00–1.01; *P* = 0.0067) and live birth (adjusted OR = 1.01; 95% CI, 1.00–1.01; *P* < 0.001), and a nonlinear correlation was observed between the E_2_/P ratio and LBR on OPU + 7.

**Conclusions:**

A higher E_2_/P ratio is associated with a higher LBR, but the E_2_/P ratio should be maintained within a suitable range.

**Supplementary Information:**

The online version contains supplementary material available at 10.1186/s13048-023-01096-3.

## Introduction

The window of implantation (WOI) is a limited timeframe in which the endometrium becomes receptive to the competent embryo for implantation. In a natural cycle, the WOI is open during the mid-luteal phase, which is driven by the sequential actions of estradiol (E_2_) and progesterone (P). Notably, different definitions of the time of implantation in terms of the time of human chorionic gonadotropin (hCG) appearance in maternal urine have been used, and they include 8 to 10 days after ovulation [1], days 7–9 after the urine luteinizing hormone (LH) surge (LH + 7–9) [2], and day 7 after the urine or serum LH peak (LH + 7) [3, 4]. It appears that no consensus has been reached on the definition of WOI.

The levels of E_2_ and P in the luteal phase reflect luteal function. During in vitro fertilization (IVF), the rise in E_2_ and P to supraphysiological levels after multiple follicles develop can adversely affect pregnancy outcomes, and to maximize the live birth rate (LBR) of an IVF cycle, it is crucial to pinpoint the WOI day. In the available literature, the time of implantation is defined as the development of pinopodes on the sixth day after the serum LH peak (LH + 6), representing the adhesion of blastocysts to the luminal epithelium [5]; as the first detection of serum hCG on embryonic days 6.6 to 7.4 (around the seventh day after oocyte pick-up (OPU + 7)) after embryo transfer (ET) [6]; or as completion of the 5^th^ day of progesterone treatment (P + 5) in the hormone-replacement therapy (HRT) cycle [7, 8]. In this context, it is noteworthy that there are controversies regarding which day of luteal E_2_ and P is most closely related to a high success rate in assisted reproduction. In our opinion, the day of OPU + 7 is closer to embryo implantation than the day of OPU + 5, therefore the levels of E_2_ or P maybe indicate a more favorable pregnancy outcome which there is a paucity of data concerning it.

To obtain data on the outcomes of women in terms of a live birth after IVF, we assessed the levels of sex hormones on OPU + 7, and then, to evaluate the influence of a significant change in the serum E_2_/P ratio on OPU + 7, we compared the effects of different E_2_/P ratios on pregnancy outcomes.

## Materials and methods

### Participants

We conducted a hospital-based cohort study. This investigation was performed in accordance with the principles of the Declaration of Helsinki and was approved by the Ethics Committee of Renmin Hospital, Hubei Medical University. Anonymous data were collected from the Reproductive Medicine Centre, Renmin Hospital, Hubei University of Medicine, between January 2016 and April 2021.

Patients who received the early-follicle-phase depot gonadotropin-releasing hormone agonist (GnRH-a) protocol were included. Patients were chosen if they satisfied all the following inclusion criteria: regular menstrual cycles ranging from 25 to 35 days; aged < 40 years; body mass index (BMI), 18–28 kg/m^2^; normal basal serum follicle-stimulating hormone (FSH) (< 10 mIU/ml) and anti-Müllerian hormone (AMH) (≥ 1.1 ng/ml) levels determined on days 2–3 of the cycle prior to controlled ovarian hyperstimulation (COH), and blastocyst transfer. The following were the exclusion criteria: patients with metabolic disorders, ovulatory dysfunction, pelvic tuberculosis, congenital uterine malformations, chromosomal abnormalities or single-gene disorders, cardiovascular diseases, or tumors. The patients were stratified into three groups based on E_2_/P ratio tertiles on OPU + 7, namely, the low group (1.3–15.7 pg/ng), middle group (15.7–28.8 pg/ng), and high group (28.8–487.2 pg/ng). Followed-up was performed by communicating with the women by telephone until the pregnancy outcomes were known.

### Ovarian stimulation

The patients received a single intramuscular injection of 3.75 mg long-acting triptorelin acetate (Decapeptyl; Ferring, SaintPrex, Switzerland) on day 2 or 3 of the cycle. After 30–42 days of downregulation, an ultrasound scan and serum concentration tests were performed, and the criteria were as follows: endometrial thickness ≤ 5 mm; follicles 5–7 mm; serum concentration of E_2_ < 50 pg/ml; P < 1 ng/ml; and LH < 1 mIU/ml. Recombinant LH (Luveris; Merck Serono) (75 IU per day) was added in the mid- and late-follicular stages to promote follicular development when the serum LH level was below 1.2 mIU/ml. Then, the treatment followed by gonadotropin (Gn) stimulation, the doses of urinary human menopausal gonadotropin (HMG, Livzon Pharmaceutical, China) and recombinant FSH (Gonal-f, Merck Serono, Germany) were adjusted according to the growth trend of the follicles and serum hormone changes (150–450 IU per day). Recombinant hCG (Merck Serono, Italy) at a dose of 250 µg and urinary hCG (Livzon Pharmaceutical, China) at a dose of 1,000–2,000 IU were given to trigger oocyte maturation when two or more follicles reached preovulatory size (18–22 mm). We chose the trigger medication when multiple follicles were greater than 16 mm in size and according to the E_2_ levels as follows: when there were more than 10 follicles and the E_2_ level was greater than 2500 pg/ml, recombinant hCG was used alone; when there were more than 10 follicles or the E_2_ level was less than 2500 pg/ml, both recombinant hCG and urinary hCG 1,000 IU were used; when there were less than 10 follicles or the E_2_ level was less than 2500 pg/ml, both recombinant hCG and urinary hCG 2,000 IU were administered. Oocyte retrieval was done 36 h following the trigger. According to the standard insemination procedures used in the laboratory, all oocytes were inseminated using IVF or ICSI. Embryo scoring was conducted based on morphologic criteria; 6–8 cells with less than 20% fragmentation were considered to be good-quality embryos. On the fifth day after oocyte pick-up, ET was carried out with a soft catheter under transabdominal ultrasound guidance.

### Luteal-phase support

After oocyte retrieval, luteal-phase support was initiated and continued daily until 3 months of gestation with the daily application of 90 mg vaginal progesterone gel (Crinone; Merck Serono) and either 10 mg twice or three times daily oral dydrogesterone (Duphaston, Abbott, USA), 2 mg twice daily oestradiol valerate tablets (Progynova, Berlin, Germany), or 1 mg:10 mg daily vaginal oestradiol and dydrogesterone tablets (Femoston, Abbott, USA). The good-quality spare embryos were cryopreserved through a vitrification protocol. Fresh ET cancellation and freeze-all strategies were implemented in cases of high P concentrations on hCG day (> 2 ng/ml) or to prevent ovarian hyperstimulation syndrome (OHSS). We chose the luteal support medication according to the P and E_2_ levels on OPU + 2: when the E_2_ level was greater than 1000 pg/ml and the P level was greater than 100 ng/ml, Crinone was used alone; when the E_2_ level was greater than 1000 pg/ml and the P level was 50–100 ng/ml, both Crinone and dydrogesterone were used; when the E_2_ level was less than 1000 pg/ml and the P level was 50–100 ng/ml, Crinone and dydrogesterone plus Progynova were used; and when the E_2_ level was less than 500 pg/ml and the P level was less than 50 ng/ml, Crinone, dydrogesterone, Progynova and Femoston were used.

### Hormone assays

We measured serum P and E_2_ levels, which represent luteal function, on OPU + 2, OPU + 5 and OPU + 7 using commercially available automated electrochemiluminescence immunoassays (UniCel® DxI 800 Access Immunoassy System, Beckman Coulter, USA and Access® Progesterone Calibrators, Access® Sensitive Estradiol Assay, Beckman Coulter, USA). Skilled technicians carried out all measurements in accordance with the manufacturer's instructions. P had a detection threshold of 0.1 ng/ml, and the in-house inter- and intra-assay coefficients of variation were 10 and 10%, respectively. E_2_ had a detection limit of 15.0 pg/ml, and the in-house inter- and intra-assay coefficients of variation were 10 and 10%, respectively.

### Outcome parameters

The outcome measures for patients with clinical and nonclinical pregnancy are presented first. Based on raw data on E_2_ and P levels throughout the early and mid-luteal stages individually, the E_2_/P ratio groups were identified.

In this study, the primary outcome was the LBR. The secondary outcomes were moderate or severe OHSS, hCG positivity, clinical pregnancy, ectopic pregnancy, pregnancy loss and preterm birth rates. Moderate or severe OHSS was diagnosed in women who fulfilled more than one of the following criteria: clinical ascites, hydrothorax, or dyspnoea (exertional or at rest) [9]. Biochemical pregnancy was defined as hCG > 10 mIU/ml 14 days after ET. Clinical pregnancy was defined as an intrauterine gestational sac identified by ultrasonography 30 days after ET. Early pregnancy loss was defined as spontaneous pregnancy loss before 12 weeks. Live birth was considered when a living fetus was born after 28 weeks of pregnancy.

### Statistical methods

The statistical packages R (The R Foundation; http://www.r-project.org; version 3.6.1), EmpowerStats (http://www.empowerstats.com) and SPSS 22.0 (IBM, Armonk, NY, USA) were utilized for all analyses. One-way analysis of variance or the Kruskal–Wallis test was used to examine the differences among groups, and continuous variables are shown as the mean with standard deviation or the median with interquartile range. Categorical variables were quantified as percentage-based figures and compared using either the Fisher's exact test or the Pearson chi-square test. Statistical significance was accepted as a two-sided *P* value < 0.05. Graphs were generated by using GraphPad Prism version 8.0 (GraphPad Software).

A multivariable logistic regression analysis was performed to assess significant relationships between the E_2_/P ratio on OPU + 7 and pregnancy outcomes. The variables that indicated significance in the univariate analysis at *P* < 0.10 or more and those that might have an influence on live birth were included in the multivariable model. The GraphPad program was used to generate a spline curve by plotting the trends between pregnancy outcomes and various hormone levels.

Smooth curve fitting models were created using EmpowerStats software and R-project (version 3.6.1) in order to further analyze the substantial relationships between the E_2_/P ratio on OPU + 7 and pregnancy outcomes. A two-piecewise linear regression model was also used to assess the threshold effect of the influencing factors on live birth using a smoothing function curve. The inflection point is obtained by recursive algorithm. Additionally, the one-line linear regression model and the two-piecewise linear regression model were also compared using a log-likelihood ratio test, and odds ratios (ORs) and 95% confidence intervals (CIs) for the threshold turning points of the independent influencing factors were computed before and after. The relationship was then further examined using model observation data, and ultimately, the chance of a live birth was properly examined using data obtained before and after each independent influencing factor's threshold inflection point.

## Results

### Luteal hormone profiles on OPU + 2, OPU + 5 and OPU + 7 of patients with clinical pregnancy and nonclinical pregnancy

The mean (± SD) patient age in this study population was 29.6 ± 3.5 years (range 18–41). After ET, 2,257 cycles (1,879 conventional IVF and 378 ICSI) produced a total of 1,606 clinical pregnancies, for a clinical pregnancy rate of 71.2%. Stratified by diagnostic classification, 1,483 patients had pelvic and tubal diseases (65.7%), 90 had endometriosis (4.0%), 470 had male factor infertility (20.8%), and 214 had unexplained infertility (9.5%) (Table 1).

**Table 1:** The baseline characteristics of the patients and the ovarian stimulation characteristics and embryological outcomes of the patients with different serum E2/P ratios on OPU + 7

Characteristic	Total	E_2_/P ratios on OPU + 7 (pg/ng)			
		Low (1.3—15.7)	Middle (15.7—28.8)	High (28.8—487.2)	*P*-value
No. of cycles	2257	752	751	754	
Female Age (years)	29.6 ± 3.5	29.8 ± 3.6	29.5 ± 3.5	29.4 ± 3.4	0.159
BMI (kg/m^2^)	23.2 ± 3.7	22.4 ± 3.5	23.0 ± 3.6	24.1 ± 4.0	< 0.001
AMH (ng/ml)	6.4 ± 3.6	5.9 ± 3.3	6.5 ± 3.5	6.7 ± 3.8	< 0.001
AFC	18.2 ± 7.1	16.8 ± 6.5	18.1 ± 7.0	19.5 ± 7.5	< 0.001
Infertility duration (years)	3.3 ± 2.4	3.5 ± 2.3	3.3 ± 2.4	3.2 ± 2.3	0.133
Infertility type					0.800
Primary	1213 (53.7%)	397 (52.8%)	409 (54.5%)	407 (54.0%)	
Secondary	1044 (46.3%)	355 (47.2%)	342 (45.5%)	347 (46.0%)	
Infertility factors					< 0.001
Pelvic and tubal factors	1483 (65.7%)	484 (64.4%)	511 (68.0%)	488 (64.7%)	
Endometriosis	90 (4.0%)	46 (6.1%)	28 (3.7%)	16 (2.1%)	
Male factor	470 (20.8%)	142 (18.9%)	151 (20.1%)	177 (23.5%)	
Unexplained	214 (9.5%)	80 (10.6%)	61 (8.1%)	73 (9.7%)	
Fertilization method					0.930
IVF	1879 (83.3%)	626 (83.2%)	628 (83.6%)	625 (82.9%)	
ICSI	378 (16.7%)	126 (16.8%)	123 (16.4%)	129 (17.1%)	
Dosage of Gn (IU)	2303.1 ± 823.4	2298.2 ± 812.0	2234.5 ± 798.1	2376.2 ± 853.7	0.004
Duration of Gn (days)	11.8 ± 1.7	11.9 ± 1.8	11.8 ± 1.7	11.7 ± 1.7	0.040
Endometrial thickness on hCG day (mm)	11.7 ± 2.6	11.5 ± 2.6	11.7 ± 2.7	11.7 ± 2.6	0.139
Moderate or severe OHSS rate	55 (2.4%)	22 (2.9%)	22 (2.9%)	11 (1.5%)	0.103
No. of oocytes retrieved	11.1 ± 2.4	10.8 ± 2.2	11.1 ± 2.5	11.3 ± 2.5	< 0.001
No. of mature oocytes	10.4 ± 2.3	10.1 ± 2.1	10.4 ± 2.3	10.6 ± 2.4	< 0.001
Fertilization rate (2PN) (%)	89.3 ± 10.0	88.6 ± 10.1	89.7 ± 10.0	89.7 ± 9.9	0.048
Cleavage rate (%)	98.9 ± 4.1	98.2 ± 5.1	99.1 ± 3.6	99.3 ± 3.3	< 0.001
No. of embryos obtained	4.8 ± 1.0	4.7 ± 1.1	4.8 ± 1.1	4.7 ± 1.0	0.751
good-quality embryo rate (%)	80.8 ± 17.0	79.9 ± 16.1	80.3 ± 17.5	82.2 ± 17.2	0.020
Blastocyst formation rate (%)	86.4 ± 19.6	83.1 ± 21.1	86.3 ± 19.8	89.7 ± 17.3	< 0.001
No. of transferred embryos, n (%)					0.026
1	1345 (59.6%)	475 (63.2%)	445 (59.3%)	425 (56.4%)	
2	912 (40.4%)	277 (36.8%)	306 (40.7%)	329 (43.6%)	
Implantation rate	65.4 ± 44.3	61.6 ± 45.5	63.8 ± 44.6	70.7 ± 42.2	< 0.001
Trigger dosage					< 0.001
rhCG 250 µg	678 (30.0%)	241 (32.0%)	231 (30.8%)	206 (27.3%)	
rhCG 250 µg + uhCG 1000 IU	195 (8.6%)	80 (10.6%)	73 (9.7%)	42 (5.6%)	
rhCG 250 µg + uhCG 2000 IU	1384 (61.3%)	431 (57.3%)	447 (59.5%)	506 (67.1%)	
Luteal support					< 0.001
C	224 (9.9%)	93 (12.4%)	73 (9.7%)	58 (7.7%)	
C + D	268 (11.9%)	68 (9.0%)	97 (12.9%)	103 (13.7%)	
C + D + P	1243 (55.1%)	403 (53.6%)	457 (60.9%)	383 (50.8%)	
C + D + P + F	522 (23.1%)	188 (25.0%)	124 (16.5%)	210 (27.9%)	

Date: mean ± SD or (%) (no./total no.).

*E*_*2*_ estradiol, *P* progesterone, *OPU* oocyte pick-up, *GnRH* gonadotropin-releasing hormone, *BMI* body mass index, *AMH* anti-Müllerian hormone, *AFC* antral follicular count, *IVF *in vitro fertilization, *ICSI* intracytoplasmic sperm injection, *Gn* gonadotropin, *hCG* human chorionic gonadotrophin, *OHSS* ovarian hyperstimulation syndrome, *PN* pronuclear number, *C* Crinone, *C* + *D* Crinone + Dydrogestrone, *C* + *D* + *P* Crinone + Dydrogestrone + Progynova, *C* + *D* + *P* + *F* Crinone + Dydrogestrone + Progynova + Femostone

**Fig. 1:**
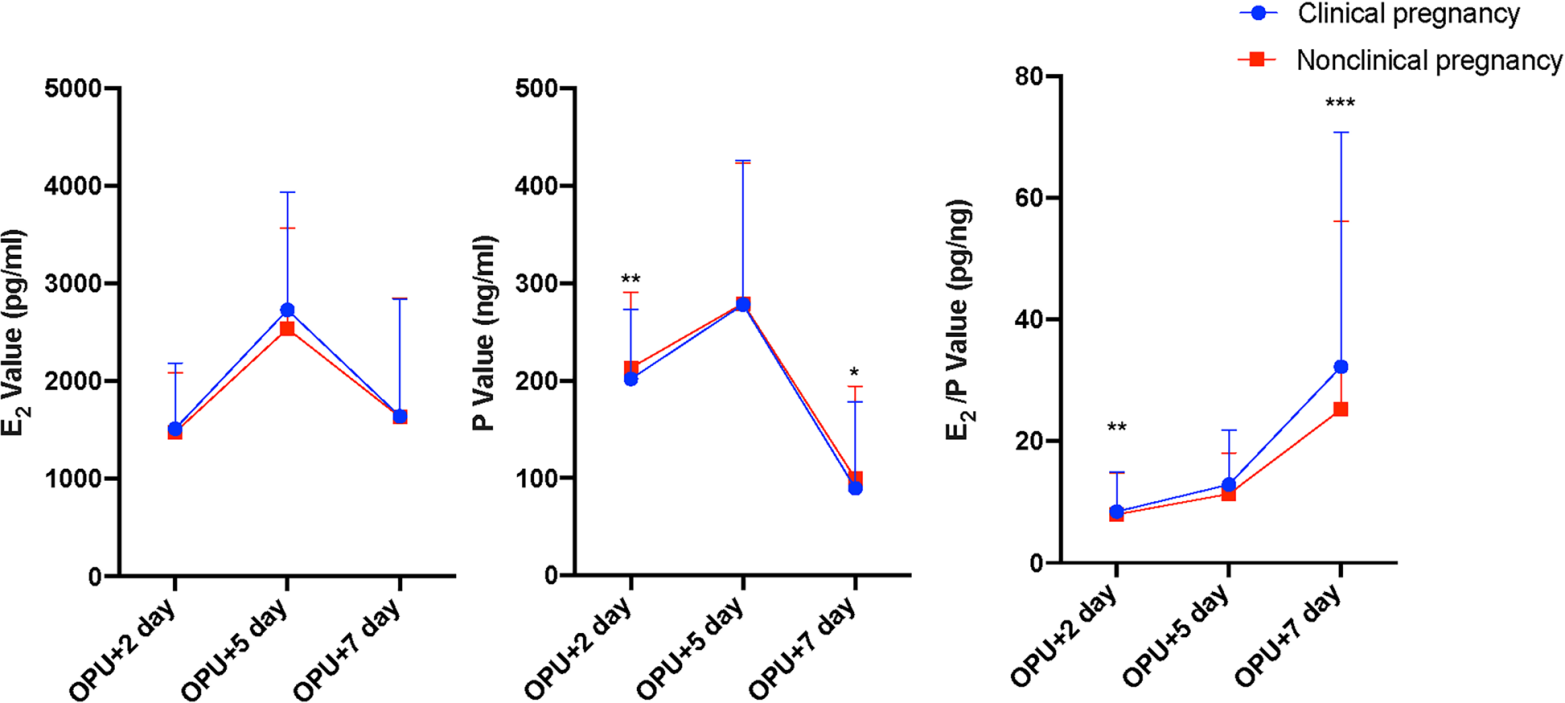
The levels of E_2_ and P and the E_2_/P ratio on OPU + 2, OPU + 5 and OPU + 7 between the groups with clinical pregnancy and nonclinical pregnancy

2,257 women who were utilizing the depot GnRH-a regimen and were split into two groups based on clinical pregnancy made up the eligible cohort. There were significant differences in BMI, AFC, trigger dosage, the moderate or severe OHSS rate, number of transferred embryos, E_2_ and P on hCG day, P and the E_2_/P ratio on OPU + 2, and P and E_2_/P ratio on OPU + 7 between the two groups (*P* < 0.05) (Supplementary Table 1), and the difference in the E_2_/P ratio on OPU + 7 was especially notable (32.3 pg/ng vs. 25.2 pg/ng, *P* < 0.001) (Fig. 1). There was no significant difference in female age, AMH, infertility duration, infertility type, infertility factors, fertilization method, dosage and duration of Gn, endometrial thickness on hCG day, number of oocytes retrieved, the good-quality embryo rate or luteal support medication between the two groups (*P* > 0.05) (Supplementary Table 1).

According to Fig. 1, the level of P in the clinical pregnancy group was lower than that in the nonclinical pregnancy group (201.9 ng/ml vs. 213.1 ng/ml, *P* = 0.001), and the E_2_/P ratio was higher than that in the nonclinical pregnancy group on OPU + 2 (8.4 pg/ng vs. 8.0 pg/ng, *P* = 0.003). The levels of E_2_ and P and the E_2_/P ratio on OPU + 5 were all higher than those on OPU + 2. On OPU + 7, the levels of E_2_ and P were lower than those on OPU + 5, but the E_2_/P ratio was higher than that on OPU + 5. Our study also revealed that the E_2_/P ratio on OPU + 7 in the clinical pregnancy group was higher than that in the nonclinical pregnancy group (32.3 pg/ng vs. 25.2 pg/ng), and the difference was statistically significant (*P* < 0.001).

### The baseline characteristics of patients, the ovarian stimulation characteristics and embryological outcomes of patients with different serum E2/P ratios on OPU + 7

The E_2_/P ratio on OPU + 7 was used to classify all patients into three groups. BMI, AMH, AFC, and infertility factors among the three groups were significantly different. (*P* < 0.05) (Table 1). Female age, infertility duration, infertility type and fertilization method did not significantly differ among the groups. (*P* > 0.05) (Table 1).

The ovarian stimulation characteristics and embryological outcomes of the three groups are also presented in Table 1. There were significant differences in the dosage and duration of Gn, number of oocytes retrieved, good-quality embryo rate, blastocyst formation rate, number of transferred embryos, trigger dosage and luteal support medication among the three groups (*P* < 0.05). There were no significant differences in endometrial thickness on hCG day or the fertilization rate (*P* > 0.05).

### Pregnancy outcomes of patients with different serum E2/P ratios on OPU + 7

The pregnancy outcomes, stratified into three groups by the serum E_2_/P ratio tertiles on OPU + 7, are presented in Table 2. There was no significant difference in the ectopic pregnancy rate, early, mid- or late-term pregnancy loss rate, preterm birth rate or number of fetuses delivered by one-way analysis of variance or the Kruskal–Wallis test (*P* > 0.05), but there were significant differences in the positive hCG rate, clinical pregnancy rate and LBR (*P* < 0.01) (Table 2). The LBRs in the three groups were 56.4%, 61.8% and 69.1% (*P* < 0.001), respectively. The positive hCG rate, clinical pregnancy rate and LBR all appeared to be significantly higher in the high-ratio group than in the low-ratio group (82.5% vs. 73.4%, 76.4% vs. 66.8%, 69.1% vs. 56.4%, *P* < 0.001).

**Table 2:** Reproductive outcomes of women with different serum E_2_/P ratios on OPU + 7

Characteristic	Total	E_2_/P ratios on OPU + 7 (pg/ng)			
		Low (1.3—15.7)	Middle (15.7—28.8)	High (28.8—487.2)	*P*-value
Positive hCG rate (%)	1748 (77.4%)	552 (73.4%)	574 (76.4%)	622 (82.5%)	< 0.001
Clinical pregnancy rate (%)	1606 (71.2%)	502 (66.8%)	528 (70.3%)	576 (76.4%)	< 0.001
Ectopic pregnancy rate (%)	12 (0.7%)	8 (1.6%)	3 (0.6%)	1 (0.2%)	0.006
Early pregnancy loss rate (%)	135 (8.4%)	56 (11.2%)	39 (7.4%)	40 (6.9%)	0.006
Mid- and late-term pregnancy loss rate (%)	50 (3.1%)	14 (2.8%)	22 (4.2%)	14 (2.4%)	0.006
Preterm birth rate (%)	256 (15.9%)	65 (12.9%)	85 (16.1%)	106 (18.4%)	0.058
Live birth rate (%)	1409 (62.4%)	424 (56.4%)	464 (61.8%)	521 (69.1%)	< 0.001
Fetuses delivered, n (%)					0.092
single	1049 (74.6%)	332 (78.3%)	342 (73.7%)	375 (72.3%)	
twins	358 (25.4%)	92 (21.7%)	122 (26.3%)	144 (27.7%)	

Date: mean ± SD or (%) (no./total no.).

*E*_*2*_ oestradiol, *P* progesterone, *OPU* oocyte pick-up, *hCG* human chorionic gonadotrophin

To accounting for potential confounders, multivariable regression analysis was used. After controlling for female age, BMI, AMH, AFC, infertility duration, infertility type, infertility factors, fertilization method, administration on trigger day, luteal support, number of transferred embryos and the moderate or severe OHSS rate, the E_2_/P ratio on OPU + 7 was positively associated with positive hCG (adjusted OR = 1.01; 95% CI, 1.01–1.02; *P* < 0.0001), clinical pregnancy (adjusted OR = 1.01; 95% CI, 1.00–1.01; *P* = 0.0067) and live birth (adjusted OR = 1.01; 95% CI, 1.00–1.01; *P* < 0.001) (Table 3). Furthermore, there were no significant differences in the ectopic pregnancy rate, early, mid- or late-term pregnancy loss rate, preterm birth rate or number of fetuses delivered after multivariable regression analysis (*P* > 0.05).

**Table 3:** Analysis of the correlation between the E_2_/P ratio on OPU + 7 and pregnancy outcomes using multivariable regression analysis

Pregnancy Outcomes	Non-adjusted	Adjust
Positive hCG	1.01 (1.01, 1.02) < 0.0001	1.01 (1.01, 1.02) < 0.0001
Clinical pregnancy	1.01 (1.00, 1.01) < 0.0001	1.01 (1.00, 1.01) 0.0066
Ectopic pregnancy	0.95 (0.90, 1.00) 0.0552	0.97 (0.92, 1.02) 0.2601
Early pregnancy loss	0.99 (0.99, 1.00) 0.0697	0.99 (0.99, 1.00) 0.0559
Mid- and late-term pregnancy loss	0.99 (0.98, 1.01) 0.2607	0.99 (0.97, 1.00) 0.0684
Preterm birth	1.00 (1.00, 1.01) 0.0075	1.00 (1.00, 1.01) 0.0331
Live birth	1.01 (1.00, 1.01) < 0.0001	1.01 (1.00, 1.01) 0.0002

Data was shown as OR (95%CI) *P* value

Non-adjusted model adjust for: None

Adjust model adjust for: Female Age; BMI; AMH; AFC; Infertility duration; Infertility type; Infertility factors; Fertilization method; Administration on trigger day; Luteal support; No. of transferred embryos; Moderate or severe OHSS rate

**Fig. 2:**
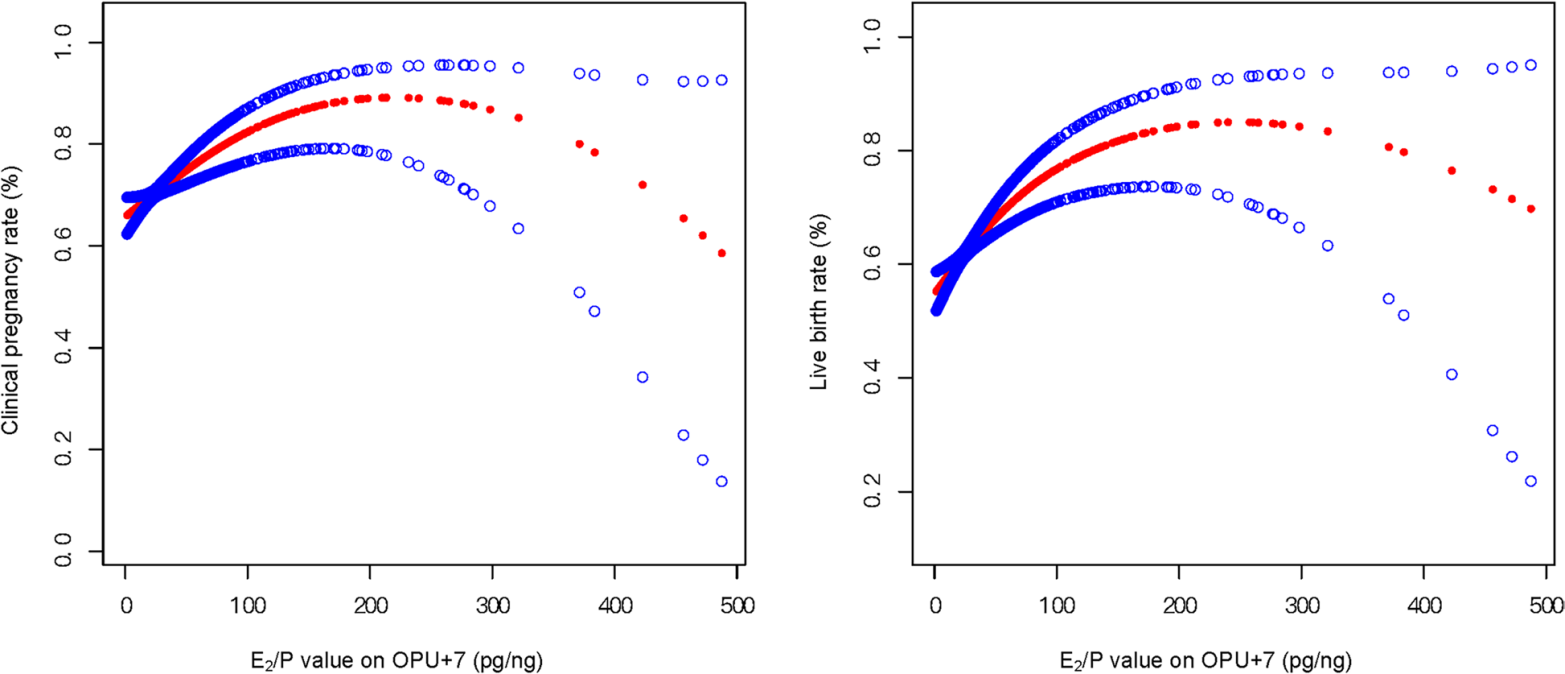
The correlation between the E_2_/P ratio on OPU + 7 and clinical pregnancy and live birth rates. A threshold, nonlinear association between pregnancy outcomes and the E_2_/P ratio on OPU + 7 day was found in a generalized additive model (GAM). The smooth curve fit between variables is represented by the solid red line. The 95% confidence interval from the fit is represented by blue bands. All estimates are adjusted for female age, BMI, AMH, AFC, infertility duration, infertility type, infertility factors, fertilization method, administration on trigger day, luteal support, number of transferred embryos and moderate or severe OHSS rate. E_2_, estradiol; P, progesterone; OPU, oocyte pick-up

A nonlinear association between the E_2_/P ratio and pregnancy outcome was revealed by the adjusted smooth curve fit. Specifically, the E_2_/P ratio on OPU + 7 had a positive correlation with clinical pregnancy and live birth. Further threshold impact research was needed, nevertheless, as these variables did not have a simple linear relationship (Fig. 2). Thus, threshold saturation effect analysis of the association between the E_2_/P ratio on OPU + 7 and clinical pregnancy or live birth was performed. The logarithmic likelihood ratio test showed that the E_2_/P ratio on OPU + 7 had a curvilinear association with clinical pregnancy and live birth and that there were two separate points (K_1_ = 78.09, K_2_ = 76.97) (*P* < 0.05). When the E_2_/P ratio was < 78.09 pg/ng (K_1_ < 78.09), it was positively correlated with clinical pregnancy (OR = 1.01, 95% CI: 1.00–1.02, *P* < 0.001). Conversely, there was no correlation between the E_2_/P ratio on OPU + 7 and clinical pregnancy rate when the E_2_/P ratio was > 78.09 pg/ng (K_1_ > 78.09) (OR = 1.00, 95% CI: 1.00–1.01, *P* = 0.94). Additionally, when the E_2_/P ratio was < 76.97 pg/ng (K_2_ < 76.97), it was positively correlated with live birth (OR = 1.01, 95% CI: 1.01–1.02, *P* < 0.001). Conversely, there was no correlation between the E_2_/P ratio on OPU + 7 and LBR when the E_2_/P ratio was > 76.97 pg/ng (K_1_ > 76.97) (OR = 1.00, 95% CI: 1.00–1.00, *P* = 0.89) (Supplementary Table 2).

## Discussion

This study, to the best of our knowledge, is the first to unequivocally show that the serum E_2_/P ratio on OPU + 7 which maybe is the day closest to the time of embryo implantation, but not OPU + 5, the day of blastocyst transfer, can be regarded as an indicator for higher LBR after IVF treatment.

Multiple studies evaluated a mean time interval of 33.91 h between the onset of the LH surge and ovulation [10] and found that the seventh day after the onset of the LH surge (LH + 7) is crucial for embryo implantation. It is noteworthy that the moment of ovulation in the natural cycle is equivalent to the time of OPU in IVF, and LH + 7 is equal to day 7 after hCG administration (around OPU + 5.5 and the day of blastocyst transfer). In this investigation, our hypothesis is that the levels of sex hormones on OPU + 7 could presage successful nidation of embryos because the embryos end their wandering time and are in the process of implantation in the uterine cavity.

During normal pregnancy, maternal plasma E_2_ levels dramatically increase from the luteal phase to the end of pregnancy. Two trials, which specifically included women on the day of ET, showed no effect of luteal E_2_ on reproductive outcomes [11, 12]. However, in previous trials involving women with higher E_2_ levels from OPU + 6 to OPU + 7, although the threshold value of the E_2_ level is nonuniform [13, 14] and variable in the daytime [15], the ongoing pregnancy rate after ET during the IVF cycle was superior to that in patients with lower E_2_ levels in the IVF cycle due to the possibility that E_2_ activation may result in angiogenic and vasodilative changes that affect implantation and regulate fetoplacental blood flow [16]. Otherwise, persistently low levels of E_2_ may have detrimental effects on placentation, therefore resulting in preeclampsia [17]. We found no differences in E_2_ levels in the luteal phase on OPU + 2, OPU + 5 and OPU + 7 between pregnant and nonpregnant women. It appears that a reasonably steady E_2_ level is linked to healthy corpus luteum activity, making it ineffective to predict endometrial receptivity from a single luteal E_2_ measurement.

The attainment of decidualization and embryo-endometrial adhesion for successful implantation are related to adequate P levels. In an intrauterine insemination trial, the mid-luteal P concentration could predict treatment failure after COH when it was lower than 25 nmol/L on the 7^th^ day after the hCG trigger (hCG + 7) [18]. Using mifepristone, a progesterone receptor modulator, altered the transcriptome associated with endometrial receptivity on day 2 after the uterine LH surge (LH + 2) renders the endometrium nonreceptive on LH + 7 [19]. These results are consistent with the importance of mid-luteal P. It is interesting to note that the peak P concentrations between 10 and 15 ng/ml in urine that occur from LH + 7 to LH + 9 appear to be sufficient to induce endometrial receptivity [20], and the maximum pregnancy rate is achieved with serum luteal P levels of 150–250 nmol/l on OPU + 5 in fresh IVF cycles [21]. Therefore, serum P levels that are too low or too high in the mid-luteal phase might reduce the likelihood of a live birth. In addition, these studies focused on the time before nidation, rather than on OPU + 7, which might be closer to the WOI. In our trial, the results favor the use of the serum E_2_/P ratio on OPU + 7 for predicting a higher LBR; specifically, the results suggest that when the E_2_ level remains steady, it is better to maintain a relatively low P level, as a higher P level does not improve the outcome.

The corpus luteum produces E_2_ and P, which are affected on OPU + 5 by the various combinations and variable doses of trigger administration and the luteal-phase support medication [22], and luteal function might be reflected by the rate of moderate or severe OHSS and pregnancy loss [23]. Regarding the above considerations, adjustments were made for these variables in our trial, and we found that in a proper range (when the E_2_/P ratio was < 76.97 pg/ng), the serum E_2_/P ratio on OPU + 7 was positively related with the rates of clinical pregnancy and live birth.

The findings of this study on mid-luteal steroid levels on OPU + 7 will be helpful for achieving a successful pregnancy and live birth. The key strengths of this study include a unified stimulation protocol that rules out the impacts of different protocols and a focus on blastocyst transfer so that the influence of embryos at different stages of pregnancy outcomes would be eliminated. Our study has several limitations. We did not have data on embryo aneuploidy, so we could not exclude the impact of aneuploidy on reducing the possibility of conception or on the serum hCG concentration on OPU + 7 to clearly evaluate whether the secretion of placental villi after embryo implantation causes hormone fluctuations. In addition, the number of patients on OPU + 5 was very small. Thus, the results may not be widely applicable to women with all stimulation protocols or frozen ET cycles. In the recent years of research, psychological variables of couples undergoing assisted reproductive technology [24], cryptic sperm defects [25], intrauterine injections of embryo culture supernatant before embryo transfer [26] and Inositol used during ovulation promotion [27–30] have also been found to have effect on pregnancy outcomes, we also believe that more relevant variables should be introduced into the further study.

## Conclusion

A higher E_2_/P ratio on OPU + 7 in fresh blastocyst transfer cycles is associated with better pregnancy outcomes, but it should be maintained within a suitable range. More well-designed randomized controlled trials are needed to examine how to optimize the individual luteal-phase support and determine the optimum starting time for luteal support.

## Supplementary Information

**Additional file 1: Supplementary Table 1.** Luteal hormone profiles at different time points between clinical pregnancy and nonclinical pregnancy patients.

**Additional file 2: Supplementary Table 2.** Analysis of the threshold saturation effect between the E2/P ratio on OPU+7 and pregnancy outcomes.

## Data Availability

All data presented in this study are available upon request upon contact with the corresponding author.
