# Supplementary material for: The serum oestradiol/progesterone ratio on the day of OPU + 7, but not the day of OPU + 5, affects the rates of live birth in fresh blastocyst embryo transfer cycles
Source: J Ovarian Res. 2023 Jan 7;16:4. doi: 10.1186/s13048-023-01096-3 (PMC9826588; doi:10.1186/s13048-023-01096-3)
Supplement: Supplementary file 1 — Additional file 1: Supplementary Table 1. Luteal hormone profiles at different time points between clinical pregnancy and nonclinical pregnancy patients. [file 13048_2023_1096_MOESM1_ESM.docx]

**Supplementary Table 1 Luteal hormone profiles at different time points between clinical pregnancy and** **nonclinical pregnancy patients.**

| Characteristic | N | Clinical pregnancy | | |
| --- | --- | --- | --- | --- |
|  |  | Yes | No | *P*-value |
| No. of cycles | 2257 | 1606 | 651 |  |
| Female Age (years) | 2257 | 29.5 ± 3.4 | 29.7 ± 3.6 | 0.151 |
| BMI (kg/m^2^) | 2255 | 23.4 ± 3.8 | 22.6 ± 3.7 | <0.001 |
| AMH (ng/ml) | 2103 | 6.4 ± 3.6 | 6.3 ± 3.5 | 0.411 |
| AFC | 2257 | 18.4 ± 7.2 | 17.7 ± 7.0 | 0.040 |
| Infertility duration (years) | 2250 | 3.3 ± 2.3 | 3.5 ± 2.4 | 0.053 |
| Infertility type | 2257 |  |  | 0.568 |
| Primary |  | 857 (53.4%) | 356 (54.7%) |  |
| Secondary |  | 749 (46.6%) | 295 (45.3%) |  |
| Infertility factors | 2257 |  |  | 0.470 |
| Pelvic and tubal factors |  | 1041 (64.8%) | 442 (67.9%) |  |
| Endometriosis |  | 63 (3.9%) | 27 (4.1%) |  |
| Male factor |  | 347 (21.6%) | 123 (18.9%) |  |
| Unexplained |  | 155 (9.7%) | 59 (9.1%) |  |
| Fertilization method | 2257 |  |  | 0.801 |
| ICSI |  | 271 (16.9%) | 107 (16.4%) |  |
| IVF |  | 1335 (83.1%) | 544 (83.6%) |  |
| Trigger dosage | 2257 |  |  | 0.008 |
| rhCGα 250µg |  | 463 (28.8%) | 215 (33.0%) |  |
| rhCGα 250µg + uhCG 1000IU |  | 127 (7.9%) | 68 (10.4%) |  |
| rhCGα 250µg + uhCG 2000IU |  | 1016 (63.3%) | 368 (56.5%) |  |
| Dosage of Gn on starting day (IU) | 2257 | 128.1 ± 25.4 | 126.3 ± 22.0 | 0.118 |
| Dosage of Gn (IU) | 2257 | 2317.4 ± 836.8 | 2267.7 ± 788.7 | 0.194 |
| Duration of Gn (days) | 2257 | 11.7 ± 1.7 | 11.9 ± 1.8 | 0.079 |
| Endometrial thickness on hCG day(mm) | 2257 | 11.7 ± 2.6 | 11.6 ± 2.7 | 0.456 |
| Hormone profiles on hCG day |  |  |  |  |
| FSH (mIU/mL) | 2248 | 14.9 ± 5.0 | 15.4 ± 5.1 | 0.027 |
| LH (mIU/mL) | 2227 | 1.0 ± 0.6 | 1.0 ± 0.7 | 0.681 |
| E_2_ (pg/ml) | 2250 | 2730.4 ± 1095.4 | 2856.8 ± 1121.3 | 0.014 |
| P (ng/ml) | 2251 | 0.8 ± 0.4 | 0.9 ± 0.4 | <0.001 |
| E_2_/P (pg/ng) | 2245 | 4178.5 ± 2852.9 | 4041.5 ± 2939.8 | 0.307 |
| Hormone profiles on OPU+2 day |  |  |  |  |
| E_2_ (pg/ml) | 2252 | 1513.2 ± 675.4 | 1478.8 ± 610.6 | 0.261 |
| P (ng/ml) | 2251 | 201.9 ± 71.6 | 213.1 ± 77.6 | 0.001 |
| E_2_/P (pg/ng) | 2245 | 8.4 ± 6.5 | 8.0 ± 6.8 | 0.003 |
| Hormone profiles on OPU+5 day |  |  |  |  |
| E_2_ (pg/ml) | 279 | 2731.7 ± 1201.7 | 2538.8 ± 1026.8 | 0.225 |
| P (ng/ml) | 279 | 277.9 ± 147.6 | 279.2 ± 143.8 | 0.948 |
| E_2_/P (pg/ng) | 279 | 12.9 ± 8.9 | 11.4 ± 6.7 | 0.183 |
| Hormone profiles on OPU+7 day |  |  |  |  |
| E_2_ (pg/ml) | 2257 | 1642.9 ± 1201.7 | 1635.3 ± 1211.2 | 0.893 |
| P (ng/ml) | 2257 | 89.5 ± 88.5 | 99.5 ± 94.9 | 0.018 |
| E_2_/P (pg/ng) | 2257 | 32.3 ± 38.5 | 25.2 ± 31.0 | <0.001 |
| No. of oocytes retrieved | 2257 | 11.1 ± 2.4 | 11.0 ± 2.5 | 0.790 |
| No. of mature oocytes | 2257 | 10.4 ± 2.3 | 10.3 ± 2.3 | 0.447 |
| Fertilization rate (2PN) (%) | 2257 | 89.6 ± 9.8 | 88.7 ± 10.3 | 0.077 |
| Cleavage rate (%) | 2257 | 98.9 ± 4.0 | 98.8 ± 4.3 | 0.314 |
| No. of embryos obtained | 2257 | 4.8 ± 1.1 | 4.7 ± 1.0 | 0.068 |
| Good-quality embryo rate (%) | 2256 | 81.1 ± 16.9 | 79.9 ± 17.1 | 0.111 |
| Blastocyst formation rate (%) | 2255 | 88.0 ± 18.4 | 82.4 ± 21.9 | <0.001 |
| Moderate or severe OHSS rate (%) | 2257 | 54 (3.4%) | 1 (0.2%) | <0.001 |
| Luteal support | 2257 |  |  | 0.850 |
| C |  | 156 (9.7%) | 68 (10.4%) |  |
| C+D |  | 187 (11.6%) | 81 (12.4%) |  |
| C+D +P |  | 886 (55.2%) | 357 (54.8%) |  |
| C+D +P +F |  | 377 (23.5%) | 145 (22.3%) |  |
| No. of transferred embryos | 2257 |  |  | <0.001 |
| 1 |  | 894 (55.7%) | 451 (69.3%) |  |
| 2 |  | 712 (44.3%) | 200 (30.7%) |  |

Date: mean ± SD or (%) (no./total no.). N, number of cycles; BMI, body mass index; AMH, anti-Müllerian hormone; AFC, antral follicular count; ICSI, intracytoplasmic sperm injection; IVF, *in vitro* fertilization; hCG, human chorionic gonadotrophin; Gn, gonadotropin; FSH, follicle stimulating hormone; LH, luteinizing hormone; E_2_, oestradiol; P, progesterone; OPU, oocyte pick-up; PN, pronuclear number; OHSS, ovarian hyperstimulation syndrome; C, Crinone; C+D, Crinone+ Dydrogestrone; C+D +P, Crinone+ Dydrogestrone + Progynova; C+D+P +F, Crinone+ Dydrogestrone + Progynova+ Femostone.
