# Supplementary material for: The serum oestradiol/progesterone ratio on the day of OPU + 7, but not the day of OPU + 5, affects the rates of live birth in fresh blastocyst embryo transfer cycles
Source: J Ovarian Res. 2023 Jan 7;16:4. doi: 10.1186/s13048-023-01096-3 (PMC9826588; doi:10.1186/s13048-023-01096-3)
Supplement: Supplementary file 2 — Additional file 2: Supplementary Table 2. Analysis of the threshold saturation effect between the E2/P ratio on OPU+7 and pregnancy outcomes. [file 13048_2023_1096_MOESM2_ESM.docx]

**Supplementary Table** **2 Analysis of the threshold saturation effect between** **the E_2_/P ratio on OPU+7 and pregnancy outcomes.**

| Outcome | Clinical pregnancy | | Live birth | |
| --- | --- | --- | --- | --- |
|  | OR (95% CI) | *P*-value | OR (95% CI) | *P*-value |
| Model I |  |  |  |  |
| Linear regression coefficient | 1.01 (1.00, 1.01) | 0.0066*^*^* | 1.01 (1.00, 1.01) | 0.0002*^*^* |
| Model II |  |  |  |  |
| Fold points (K) of OPU+7-E_2_/P | 78.09 |  | 76.97 |  |
| < K regression coefficient 1 | 1.01 (1.00, 1.02) | 0.0009*^*^* | 1.01 (1.01, 1.02) | <0.0001*^*^* |
| > K regression coefficient 2 | 1.00 (1.00, 1.01) | 0.9400 | 1.00 (1.00, 1.00) | 0.8882 |
| Logarithmic likelihood ratio test |  | 0.036*^*^* |  | 0.003*^*^* |

**P* <0.05. Data was shown as OR (95%CI) *P* value.

Effect: Clinical pregnancy; Live birth. Cause: E_2_/P ratio on OPU+7 day.

Adjusted: female age, BMI, AMH, AFC, infertility duration, infertility type, infertility factors, fertilization method, administration on trigger day, luteal support, number of transferred embryos and moderate or severe OHSS rate.
